# Life Finds a Way: Young Adults With Lesbian Mothers Reflect on Their Childhood Prior to Legal Recognition of Same-Sex Parents in Sweden

**DOI:** 10.3389/fpsyg.2020.00690

**Published:** 2020-04-15

**Authors:** Anna Malmquist, Sandra Andersson, Julia Salomonsson

**Affiliations:** Department of Behavioural Sciences and Learning, Linköping University, Linköping, Sweden

**Keywords:** lesbian parents, female same-sex parents, young adults, legal recognition, family law

## Abstract

The strapline “life finds a way,” from the classic movie Jurassic Park, referred to how the all-female dinosaurs in a theme park had been able to reproduce, despite the laws of nature. Similarly, the participants in the present study described how their lesbian mothers had shown that “life finds a way,” when having children and forming a family, prior to the legal recognition of same-sex parents in Sweden. The study draws on interviews with eight young Swedish adults, aged 17–30 (average age 25). They had been raised by lesbian couples but were born prior to the legal recognition of same-sex parenthood. Prior to a legal change in 2003, a same-sex couple could not share legal parenthood. Further, female couples were excluded from Swedish assisted reproduction programs until 2005. The interviews have been analyzed thematically, and the article presents the results in four themes. The first theme, *circumvent, oppose, or adapt to legal obstacles*, shows the participants’ reflections on how their parents navigated legal obstacles in order to have children and to live together as a family. The second theme, *legal obstacles do not affect everyday life*, depicts a common experience of how a lack of legal recognition seldom mattered to the participants during their childhood. Rather, they explained how their parents had been able to form parenthood and close relations without legal recognition. In contrast, the third theme describes *occasions when legal parenthood matters*. This theme highlights occasions when the lack of legal parenthood was problematic or devastating for the participants, such as when parents divorced, or one parent died. The final theme, *the meaning of legal parents in adulthood*, explores the participants’ reflections on the meaning and impact of legal ties (or lack of legal ties) between themselves as young adults and their parents. The findings are discussed in relation to previous research on children and young adults with same-sex parents.

## Introduction

This article focuses on Swedish young adults whose lesbian mothers made huge efforts to be able to have and raise children, because of legal and social constraints, including lack of access to assisted reproduction. Parallel with changing societal attitudes, reformed legislation and technical advances in assisted reproduction, parenting has become more accessible to those living outside of heterosexual normativity (Evertsson et al., forthcoming; Golombok, 2015; Reczek, 2020). However, the participants in this study were born before many of these changes came into force. Their parents had to seek paths to parenthood outside public health care and gained no legal recognition of the non-biological mother’s parenthood. These families’ experiences of and perspectives on parenthood and origin are important to capture in order to understand the constraints that heteronormative attitudes and exclusionary legislation may create. Further, their narratives shed light on how people find ways to form a family and practice parenting despite obstacles. Contemporary Swedish legislation allows same-sex couples to share legal parenthood (Mägi and Zimmerman, 2015). Similar legal recognition has been established in many Western countries but remains uncommon in large parts of the world (ILGA, 2019). For example, same-sex parents in most Eastern European countries today experience similar lack of recognition and may experience problems similar to those described in the present study (Štambuk et al., 2019).

Previous research on lesbian families has had a major focus on the parents’ experiences and perspectives (for overviews, see Golombok, 2015; Reczek, 2020). However, the views and perspectives of offspring may not always correspond to those of parents. The present study is concerned with the perspectives of young adults with lesbian mothers and focused on their experiences of and reflections on family legislation in relation to their family of origin. First, we describe how family law on same-sex parenting has changed during the past decades in Sweden. Thereafter we will provide a brief overview of previous research on offspring in lesbian families, followed by an overview of offspring views of parenting and family in general.

### Lesbian Families in Swedish Family Law

Female same-sex parenting couples were for a long time unrecognized by Swedish law (Malmquist, 2015; Mägi and Zimmerman, 2015). Marriage and assisted reproduction treatments were exclusively reserved for different-sex couples, and adoption was only allowed for single people and married couples. In 1995 male and female same-sex couples earned the right to register their partnership. The partnership law can be described as a copy-paste of the marriage law – with two major exceptions, namely that registered partners were explicitly excluded from both fertility treatment and adoption. In the high-pitched debate that preceded the law, opponents of the registered partnership law expressed their fears that if gays and lesbians were allowed to ‘marry’, they would claim rights to parenting too (Malmquist and Zetterqvist Nelson, 2008). Such rights were indeed soon claimed by LGBTQ organizations, and a public investigation was set out in 1999 to investigate the possibility of inclusive legislation on adoption (SOU, 2001:10). Following the recommendations of the investigation, registered partners gained the right to apply for joint adoption and second-parent adoption in 2003. This legal change enabled, for the first time, Swedish same-sex couples to share legal parenthood and guardianship. Joint adoptions have remained uncommon among Swedish same-sex couples (Malmquist and Spånberg Ekholm, 2020), but second-parent adoption was utilized primarily by lesbian couples who had had children though self-insemination or at fertility clinics abroad (Malmquist, 2015). Two years later, in 2005, female same-sex couples were given access to assisted reproduction treatment through the Swedish health care system, and in 2009 a gender-neutral marriage law replaced the registered partnership law.

The present Swedish law enables most lesbian couples to share legal parenthood of their children. The birth mother is automatically registered as legal parent at birth, while the non-birth mother can be established as legal parent through a signed confirmation or second-parent adoption.

Lesbian couples’ pathways to parenthood have changed in parallel with the law. Those who had children in the 1990s and early 2000s most often conceived through self-insemination, as this was one of few options available (Zetterqvist Nelson, 2007; Ryan-Flood, 2009). Unlike lesbian women in many other western countries, Swedish women often chose to involve a man or a male couple as fathers, rather than simply use a donor. Since lesbian couples gained access to second-parent adoption (in 2003) and fertility treatment in public health care (in 2005), the number of children born into lesbian-led families has grown steadily (Evertsson et al., forthcoming). Conceiving at a fertility clinic, and raising children in a two-mother-family without a father, has become by far the most common route to having a child among lesbian couples (Malmquist, 2016). In Swedish law, a child can only have two legal parents, a limitation that leaves remaining legal obstacles for families formed with more than two plausible parents (Malmquist and Spånberg Ekholm, 2020). Thus, the two-mother-family is now legally recognized, while families with more than two social parents are not.

### Offspring in Lesbian Families

Research exploring lesbian family forms has its origins in the late 1970s and has expanded steadily thereafter (Golombok, 2015). Early studies in the field commonly engaged with the question of the children’s psychological outcome (Golombok et al., 1983, 1997). Offspring outcomes were measured in terms of psychological wellbeing, social skills, gender conformity, etc., and were compared to standardized test norms or those of matched groups of children with heterosexual parents. Early studies often concerned children born within heterosexual marriages, who came to grow up in a lesbian family when their parents divorced and the mother entered a same-sex relationship. Later studies have often focused on children born into a lesbian family, as a result of assisted reproduction (e.g., Bos and Van Balen, 2008; Gartrell et al., 2019). Generally, results from offspring outcome studies show that differences between those raised by same-sex and different-sex parents are few and small (Biblarz and Stacey, 2010).

Some researchers have turned their interest to offspring in lesbian families, not to measure their psychological outcome, but to capture their narratives. Several studies have focused on experiences of openness, disclosure, and stigmatization among young people with lesbian parents (e.g., Epstein et al., 2013; Lick et al., 2013; Kuvalanka et al., 2014; van Rijn-van Gelderen et al., 2015; Cocker et al., 2019). Experiences of stigmatization are not uncommon among children in lesbian families, and studies delineate how they find strategies to respond to prejudicial or diminishing comments (Epstein et al., 2013; van Rijn-van Gelderen et al., 2015). However, children in lesbian families seem no more exposed to bullying compared to other children (Vanfraussen et al., 2002). Another explored issue has been images of and curiosity about anonymous sperm donors (Goldberg and Allen, 2013; Slutsky et al., 2016; Zadeh et al., 2018). It has been shown that offspring tend to hold far more negative attitudes than their parents toward anonymous gamete donation (Skoog Svanberg et al., 2019). Most studies of offspring in LGBTQ families include adolescents and/or young adults as participants, but some studies have focused on younger children (Tasker and Granville, 2011; Malmquist et al., 2014; Frisk Kockum and Grönbäck, 2018).

Two previous studies have focused on the situation of lesbian families in Sweden prior to legal recognition, from an offspring perspective (Zetterqvist Nelson, 2001; Nordén, 2018). The earliest study focused on the perspectives of young people who had experienced one of their parents coming out as lesbian or gay post-divorce (Zetterqvist Nelson, 2001). The study focused on the participants’ feelings about the parent’s un-normative sexuality and indicated predominantly positive attitudes and good parent-child relations within families. The second study focused on young adults who had grown up with LGBT-parents prior to legal recognition (Nordén, 2018). Most of the participants had been conceived within a heterosexual relationship, while some had been born as a result of a planned lesbian and/or gay family. The study focused on the participants’ experiences of school and leisure time, showing how heteronormativity gave raise to different kinds of social conflicts in relation to teachers, classmates, and friends, with such conflicts sometimes becoming a major issue for the children and had an impact on their education. None of these previous studies focused directly on how legislation affected the families.

### Offspring Views on Parenting and Family

Research on parenting and family has over decades been conducted within a range of disciplines, e.g., anthropology, sociology, and psychology (James, 1999). However, most studies have primarily investigated parenting from a parent’s perspective (Dannesboe, 2016). The lack of offspring perspective on family and parenthood has often been motivated by the opinion that children’s stories are incomplete and unreliable (Pascal and Bertram, 2009; Dannesboe, 2016). Children have not been seen as actors in parent-child relationships in the same way as parents have thus, parents have been considered more suitable as informants to understand parenting (Bäck-Wiklund and Bergsten, 1997; James, 1999). When the perception of the child’s role in the family changed – from a passive figure formed by the parents’ care, to an active agent who, together with the parents, creates family life – offspring experiences of parenthood have gained more attention in parental and family research (Bäck-Wiklund and Bergsten, 1997). Similarly, the understanding of the concept of “family” has changed to become more dynamic, which has meant a shift in the focus of family research from “being a family” to “doing family” (Morgan, 2011). All family members, including the children, contribute to the “doing” of family. In the expanding research on parenting from an offspring perspective, it has been shown that children see themselves as active actors in parenting and family (James, 1999). Children talk about parenting as a reciprocal process in which they are active agents (Rigg and Pryor, 2007; Tinnfält et al., 2015).

How offspring conceptualize family can be expected to change over time, both on a societal level (as discourses on families change) and on an individual level (as cognitive and socioemotional skills develop, and personal experiences change over time). Studies have shown that younger children emphasize cohabitation and shared activities in their definitions of family, whereas teenagers and young adults define family more on the basis of emotional ties (Andersson and Högstedt, 2002; Anyan and Pryor, 2002).

Researchers have also identified how variation in the conceptualization of family is related to an individual’s own experiences of family. Those who have experienced a transformation in their family, or grown up in a non-normative family, tend to have a broader definition of what a family is, in that their image of family is somewhat more likely to include family members they do not have genetic ties to, as well as family members they do not share housing with (Anyan and Pryor, 2002; Bergcrona and Krantz, 2014).

#### The Present Study

In 2018, a public investigation suggested that Swedish family law should introduce a “parental assumption” for the female the spouse of a birth parent, equivalent to the paternity assumption valid for male spouses of birth mothers (i.e., that the female spouse of a birth parent would be registered as legal parent and guardian automatically). This suggestion has not yet been legislated. As part of the investigation that foregone the suggestion, AM, a researcher within the field of same-sex parenting, was requested to do an interview study on children, youths and young adults who had grown up with lesbian mothers and/or been conceived through gamete donation. The study aimed to provide knowledge of the offspring’s perspective on family formation, genetics, and legal parenthood. A full report of the results from this study was published as an appendix to the public investigation (Malmquist, 2018) and as two master dissertations in psychology (Andersson and Salomonsson, 2018; Frisk Kockum and Grönbäck, 2018).

The present article explores in more detail a limited part of this collected data, focusing on the adult participants’ (i.e., those over 17 years of age) reflections concerning their family of origin in relation to family legislation. The aim of the article is to explore how young adults with lesbian mothers have experienced legal restrictions and obstacles encountered in their family of origin.

## Materials and Methods

The present article presents findings from interviews with eight young people (aged 17–30 years) who grew up with lesbian mothers. These interviews were conducted as part of a larger data collection in which a total 18 children, adolescents, and young adults participated. All participating offspring in the study were conceived by gamete donation and/or had grown up with same-sex parents. Two different studies were designed. The first study had a focus on children and adolescents (aged 10–15 years), and the second on adolescents and adults (those above 16 years of age). Both studies were approved by the regional ethics board at Linköping University and were funded by the public investigation that had commissioned them.

### Procedure

Participants were recruited through advertising in social media forums aimed at LGBTQ families, children of LGBTQs and people conceived through gamete donation. Those who contacted the researchers were sent an information letter describing the aim of the study and the interview procedure. The letter emphasized that participants would remain anonymous, and that they had the option to withdraw from the study at any time.

A total of 15 young adults contacted the researchers. Three of these young adults decided not to participate after receiving the information letter, and a further two did not participate due to problems scheduling an interview. In total ten participants, aged 17–32, were interviewed. Of the interviewed group two were conceived by donor insemination to heterosexual couples, thus the remaining eight, who had grown up with lesbian mothers, are the focus of the present analysis.

### Participants

Five of the eight participants identified as men and three as women. All lived in, or near to, larger cities in Sweden. Four of them were full-time students, one studied part-time and worked part-time, and the other three were employed. Four participants reported high school as their highest finished education and two had finished tertiary studies. One was currently in high school and one had finished high school without completing grades.

The participants were born between 1988 and 2001. Two participants had grown up with two mothers and had no contact with their respective sperm donors. Two participants had been raised by two mothers but had had a close relationship with their sperm donor and described him as their father. One participant had been raised by two fathers and two mothers in two separate households. Three participants were born as a result of a heterosexual relationship but had been raised in a lesbian-led family after parental divorce.

### Interviews

The interviews followed a semi-structured interview guide that included questions about three main areas: *parenthood*, *knowing (or not knowing) one’s genetic origins* and *regulation of legal parenthood.* Tjora (2012) describes semi-structured interviews as a useful method for data collection to capture reflections, opinions, and experiences of specific subjects. The majority of the questions were open-ended to allow the participants to raise potentially rich new issues not previously considered (Tjora, 2012).

The interviews were conducted in places which the participants had requested or approved of following the researchers’ suggestions, e.g., libraries. SA and JL conducted the interviews. Participants were informed that they could take a break or stop the interview at any time. All interviews were audio-recorded and transcribed verbatim. The duration of the interviews varied from 30 min to 1 h and 44 min, with an average duration of 1 h and 2 min. Any identifying information such as personal names, locations, etc., was changed to maintain anonymity.

### Analysis

The transcribed data was analyzed thematically. In a thematic analysis, the researchers search for patterns within a qualitative dataset, and describe the themes they identify (Braun and Clarke, 2006). This study is based on a social constructive epistemology (Burr, 2003), as it is assumed that the participants’ understandings of family, genetic origin and legal recognition are subjective and contextualized by their social situation and personal experiences, as well as by the broader historical and social context of Swedish lesbian families. Further, the way their narratives are presented must be understood in relation to the context of an interview study.

As a first analytical step, the entire dataset was coded by Andersson and Salomonsson separately. Codes were kept close to the data and then compared across interviews, to find a high level of concurrency between interviews. Then the codes were organized into candidate themes by Andersson and Salomonsson separately. The researchers’ candidate themes were similar but not identical. The candidate themes were compared and discussed with Malmquist, until the researcher’s agreed on how to present the result. For this article, candidate themes that concerned experiences of legal restrictions and obstacles in the family of origin were selected for further analysis. In the next step, all transcripts were re-read and relevant excerpts for each candidate theme were selected by Malmquist. The candidate themes were revised by the researchers jointly during this phase until the final four themes were established.

The excerpts presented below have been edited somewhat to make them easier to read, i.e., grammar has been corrected and filler-words removed. The convention of using […] in transcripts where parts of the excerpt have been removed, has also been utilized. When the interviewers are cited, the letter “I” for “Interviewer” has been used. When participants are cited, they are represented by the first letter of their pseudonym.

## Results

Four themes reflect how participants described their thoughts and experiences of family legislation in relation to their lesbian-led family of origin: *Circumvent, oppose, or adapt to legal obstacles, Legal obstacles do not affect everyday life, Occasions when legal parenthood matters*, and *The meaning of legal parents in adulthood.*

### Circumvent, Oppose, or Adapt to Legal Obstacles

When describing how their parents were able to have and raise children, most participants acknowledged that during the period they were conceived, there were obstacles for same-sex couples to become parents. They described how their parents had searched and found a pathway to parenthood and family life, and depicted a process that had involved circumventing, opposing or adapting to the legal obstacles their parents had encountered.

At the time of the participants’ conception, female same-sex couples had no right to assisted reproductive treatment in Swedish health care, nor had they the right to adopt. Thus, self-insemination at home and fertility treatment abroad were reported as the main option considered by their parents. For example, Amanda, described the circumstances surrounding her conception and how these affected how her mothers became parents:

Yes, they decided that they wanted kids, and started looking into possibilities, even at that time I think there were possibilities to travel abroad, but [.] that possibility didn’t exist for them at least, because they had no money and no training and no jobs and nothing. So they couldn’t afford, like, to pay to get a kid. And they would never have, like, been approved for adoption or anything like that, the only way was to ask a friend. (Amanda, 30, two mothers)

According to Amanda, financial factors and legal obstacles had affected her mothers’ options for having children. Traveling to a country where assisted reproduction was accessible for female couples (e.g., Denmark) was a possibility for same-sex female couples to conceive children without the risk of a biological father being able to claim legal parenthood (Zetterqvist Nelson, 2007). However, self-insemination with the help of a friend was the only remaining option for couples who couldn’t afford such travel. Self-insemination may have been the most preferred option for some couples, but in Amanda’s narrative it was depicted as the only option, as adoption and fertility clinics were not accessible. Thus Amanda’s mothers had adapted to the legal restrictions and found a path that was feasible.

Another participant, Fredrik, described how his mothers had found strategies to circumvent legal obstacles to have children. Both his mothers had adopted children before they had Fredrik, who was born after self-insemination. Because adoption had been available for single people and different-sex couples, although not for same-sex couples, Fredrik described how his mothers had “divorced and remarried repeatedly, to be able to adopt from other countries.”

Besides having to navigate the law to be able to have children, some participants also described how their parents circumvented legislation on legal parenthood. Because Swedish law considers a private sperm donor to be the legal father, some participants described how their parents had concealed the identity of the donor from the sociolegal authorities. Amanda said:

They [the mothers] had prepared a story to tell the family court and, like, what’s it called, the Social Insurance Agency. Because they were forced to lie to them, otherwise they wouldn’t have been able to live their lives the way they wanted to. [.] there was a paternity inquiry, so she [the birth mother] went to the family court and said she had been on a cruise I think it was, and had gotten pregnant, had no idea who the father was, and then they closed it and so she was sole guardian and parent, father unknown. (Amanda, 30, two mothers)

Like Amanda’s mothers, a few participants described how their parents chose to let their child grow up with only one legal parent, rather than reveal the donor and risk him claiming legal fatherhood. This was done to avoid a donor having the right to make legal decisions about the child and can be understood as a strategy to circumvent a legal obstacle. As a same-sex spouse was not able to second-parent adopt until 2003, the non-biological mother could not become a second guardian and thus prevent the donor making parental claim. However, some participants described situations where their non-legal mother had indeed acted as a legal guardian, for example, by signing papers intended for guardians. This can be interpreted as attempting to oppose limitations that arise when a social parent raises a child without being the legal parent.

### Legal Obstacles Do Not Affect Everyday Life

As previously described, social and legal parenthood were incongruent during each participant’s childhood. Despite this obstacle, most participants claimed that this had not caused any specific problems in their everyday lives. In their experience, their non-legal parents had not been any “less” a parent because of being perceived as having performed all aspects of parenting. Some participants could not think of any situation where legal parenthood had mattered, whereas others described events in which non-legal parents had made decisions that were formally intended for legal guardians. All participants emphasized that the absence of legal ties had not led to weaker emotional or social bonds. Rather, they reported that their non-legal parents, who had been present since birth, had had just as much responsibility and authority as their legal parents. One participant, Nora, depicted legal parent status as irrelevant to her experience of family:

I: So you have actually lived, that is, most of your life with one guardian, right?

N: Yes, I mean, if you check the documents it has always been like that, I’ve always had one mom and then we’ve had another person who has been there, but it hasn’t been like that in real life, both have been my parents, equally always. (Nora, 24, two mothers)

While Nora acknowledged the formal legal differences between her mothers, she claimed that this difference did not correspond to her experience of their parenting. Rather, she had thought of them as being her parents “equally always.” Later on, Nora explained that her parents had each had an equal say in making decisions about her. Nora, and several other participants, experienced their parents as being equal decision makers, highlighting how non-legal parents can in fact exert authority over their child without having the formal rights that legal parenthood entails. It was often presented as a self-evident or taken for granted fact that a social parent had the authority regardless of legal ties.

In contrast, a participant who was born within the context of his birth mother’s heterosexual relationship, but who had grown up with his mother and her female partner, did not portray his stepmother as an authority; she was not seen as an actual parent. Rather, this participant claimed that all the parental decisions made about him were made by his parents of origin. However, when his stepmother later gave birth to a child, the participant considered his brother to be the child of both mothers. Thus, parental authority seems to depend on the social bond, and intended parenthood at birth, rather than actual legal guardianship.

Just as the participants did not experience legal parenthood as making a real distinction between their parents as they experienced this, several also thought that parents never distinguished between siblings based on legal ties. Several participants had siblings they lacked legal ties to and also again pointed out that this had not affected the social bond between them.

In addition, several participants explained that their non-legal parents had for the most part been acknowledged as parents by people outside the family. Further, several participants gave examples of situations where non-legal parents’ signatures on documents had been accepted by their school. Non-legal parents also had usually been invited to parent-teacher meetings. Patrik explained:

Everyone has probably been invited, but it has been either Louise or Hanna for now, but I had a meeting this spring when both Harald and Björn were there but not my moms, so it varies. It depends on who can make it. (Patrik, 17, two mothers and two fathers)

Patrik had four social parents and had grown up in two households. He described how practical considerations, rather that legal parenthood, determined who would participate at parent-teacher conferences. Thus, although only the biological parents were legal parents, all four had been invited to the school. The majority of the participants said that schools had been relatively forthcoming in this regard.

According to the participants’ accounts, legal obstacles to same-sex couples’ parenthood had rarely been noticeable in everyday family life. Non-biological parents were perceived as exerting an all-compassing parental influence despite not being legal parents. Some participants reflected that legal obstacles might have been problematic for their parents, even though these did not affect them as children, at least not in their everyday lives.

### Occasions When Legal Parenthood Matters

As shown in the previous theme, most participants explained that legal parenthood had little or no impact on their everyday lives when growing up. However, a few participants shared narratives where the absence of a legal tie between themselves and their non-biological parents had led to serious consequences for them on a specific occasion. These narratives concerned events where the family had been transformed, when the parents had separated or when one parent had died. Amanda described what happened after her parents broke up:

I: Did you notice at all that your biological mom was your guardian but not your other mom?

*A: No, I mean, not when I was small, I noticed it later when they*… *That is, yes, I have to say now, because I noticed it when they moved far away from each other and couldn’t have me alternating weeks as they had in the beginning. Then it became legally difficult to do anything other than split the kids up, which normally isn’t done, because legally they only had the right to one kid each, so we have grown up with one mom each, and apart from each other, which is unusual for siblings. (Amanda, 30, two mothers)*

Amanda described how her parents had moved far away from each other after their separation. In this situation Amanda and her sister had been separated from each other, and from their respective non-legal parent. The lack of legal ties had meant that her bond to one parent had been neglected, and had led to the situation where she and her sister grew up in separate homes. Amanda reported that her mothers took legal restrictions into account when solving the issue of where the children would live. Her comment “Then it became legally difficult to do anything other,” shows how legislation had limited everyday relations between family members.

In addition to separations between the mothers, legal parenthood also had been actualized in conflicts between legal and non-legal parents. Gustav explained:

*I know there was an occasion where my moms and my dad had a fight, and he [the father] insisted that at the parent-teacher meeting in school, that Oskar, my dad, and Elna [attend]. Doris was, like, she had to wait outside [*…*] I think it was hard for her [Doris]*… *um, it wasn’t hard for me, I had two parents there and I mean, meetings were never really important (Gustav, 23, two mothers and a father)*

In a conflict between the mothers and the father, Gustav described how the father had used his authority as a legal parent and forbade the non-legal mother’s participation at the parent-teacher conference. Gustav acknowledged that the event might have been hard for his non-legal mother, but stressed that it had not mattered to him, rather the issue was downplayed as he claimed that “meetings were never really important.” The narrative can be understood as a way for him as child to keep himself outside the parental conflict, by claiming that it did not reflect anything important.

For another participant, Fredrik, inheritance legislation was a reason for him to desire to be formally adopted by his non-legal mother. Fredrik described how his non-legal mother also wanted to ensure that he would be her direct heir:

When I was growing up, Vanessa expressed that, like, she would like to adopt me so that I actually would be a legal – her legal child, so there wouldn’t be any problems with inheritances and stuff when she passes away, but with my dad the inheritance has been a protracted matter as well, so there’s that. We have put it off, in order to not complicate the legal process. (Fredrik, 20, two mothers and a father)

When Fredrik was born, same-sex partners were not allowed to second-parent adopt. The law changed in 2003, when Fredrik was 5 years old, and he explained that his non-biological mother had wanted to adopt him. However, Fredrik grew up with three social parents: two mothers and a father. Since by Swedish law a person can only have two legal parents, an adoption by the non-biological mother would have erased the legal tie between Fredrik and his father. The death of his father had reopened the question, but as Fredrik explained, there had been a drawn-out inheritance dispute after his father passed away, and therefore the desired second-parent adoption had not yet occurred.

The situations of parental separations and deaths show how legal parenthood does become relevant in certain instances for people whose social and legal parenting situation does not coincide, despite the more common experience among the participants that legal parenthood had been irrelevant. These narratives further show how lack of legal ties may continue to affect participants into adulthood.

### The Meaning of Legal Parents in Adulthood

All participants explained that absence of legal parenthood had not affected their emotional relationship with their non-legal parents in adulthood. Rather than focusing on legal or genetic ties, they emphasized the meaning of social bonds, care and closeness. Fredrik stressed how his adult relationship to his parents was primarily grounded in the social bonds formed during childhood:

Then it’s much more important in my opinion that you – that you express the value of you growing up with me, in that way you are my child, and that you show that parenthood and childhood and things, are so much more than just genetics. (Fredrik, 20, two mothers and a father)

Some participants described how they felt closer to one of their parents than the other(s), but this was never attributed to legal ties, but rather to similarities in personal qualities, such as personality or a shared sense of humor. Several participants had not previously reflected on their parents’ legal status, and some of them were unaware of the fact that according to Swedish law, a person cannot have more than two legal parents. Thus, they had not realized that all their social parents were actually not their legal parents and had not been their legal guardians. Despite the fact that same-sex couples have had access to second-parent adoption since 2003, only one of the participants, Nora, had been adopted by her non-biological mother. In the quote below she reflected on what the adoption had meant to her:

*My reality agrees with, like I don’t know, how can I say it in a good way, I mean, I’ve been*… *I’ve always had my reality, it has always been, I have my moms, both are equally my parents. And now it also says so on paper so that, well like we were talking about the inheritance, I also have the right to inherit from my other mom, also things like that. And my brother and I have suddenly become proper siblings too, in that case we weren’t siblings either (laughs). (Nora, 24, two mothers)*

Nora was adopted at the age of 22 by her non-biological mother. Her experience shows how a congruence between social and legal parenthood can be validating for the young adult. The adoption reflected her reality, both in relation to her non-biological mother and to her brother. Nora had grown up with two mothers and had no contact with the sperm donor. However, more than 10 years had passed since the law allowed her non-biological mother to apply for adoption, before they eventually went through the process.

For some participants, second-parent adoption had not been actualized, because they had three or four social parents, and Swedish law admits only two legal parents (Mägi and Zimmerman, 2015). For a few participants their parents’ separation made a second-parent adoption impossible, because Swedish law only allows the spouse or cohabitant of the legal parent to adopt as a second-parent. Thus, the social family situation of these young adults will continue to be at variance with the legal situation. Although they stress the irrelevance of this, or are even unaware of the legal limitations, it must be pointed out that they will not inherit their non-legal parents unless a valid testament is legally filled. Thus, if it has not already, the lack of legal ties will become evident when they lose a parent.

## Discussion

The strapline “life finds a way,” from the classic movie Jurassic Park, referred to how the all-female dinosaurs in a theme park had been able to reproduce, despite the laws of nature (Crichton et al., 1993). Likewise, the participants in the present study showed, through their narratives of their own becoming, how “life finds a way.” Of course, their lesbian mothers did not challenge the laws of nature to have children, but they did find pathways to have and raise children despite huge obstacles presented by the laws of society.

Several participants indicated that self-insemination was the only available option for their parents to have children, since assisted reproduction treatment and adoption were not allowed for same-sex couples in Sweden at the time they were conceived (Mägi and Zimmerman, 2015). Moreover, options of going abroad for fertility treatment were limited for many, because of personal financial and practical resources. These findings echo those of previous studies of Swedish lesbian parent families from this time, where self-insemination is described generally as the most common path to parenthood, aside from having children from previous heterosexual relationships (Zetterqvist Nelson, 2007; Ryan-Flood, 2009; Nordén, 2018). Similar findings are also shown in contemporary studies of lesbian families from countries where female couples are still excluded from assisted reproduction treatment (e.g., Štambuk et al., 2019).

Self-insemination was not conducted without risks or obstacles though, as a private sperm donor could be registered as a legal parent, regardless of the wishes of the involved parties (Mägi and Zimmerman, 2015). Some participants stated that their parents had limited this risk by lying in paternity investigations. Others reported instead that their parents had chosen a donor whom they could accept as a legal parent, and some had shared a social parenthood with him as an involved father. Similar strategies have previously been shown common among lesbian parents in Sweden and elsewhere (Park et al., 2016; Côté and Lavoie, 2019). The participants’ parents had navigated the legal landscape to find pathways to have and raise children, a process that included circumventing, adapting to, and also opposing legal obstacles.

The narrative of overcoming huge obstacles to have children has also been prominent in previous studies, in which lesbian parents who had children prior to legal recognition were interviewed (Zetterqvist Nelson, 2007; Ryan-Flood, 2009). The participants of these studies described how they had encountered practical, social, and legal obstacles. They presented themselves as pioneers, and recent Swedish statistics confirm that only a few women in registered partnerships became parents at that time (Evertsson et al., forthcoming). Thus, even though it is true that “life finds a way” sometimes, it is also the case that many other lesbian couples did not have children. With changed legislation, the options open to lesbian couples have increased, and the number of married female same-sex couples who have children has grown rapidly (Evertsson et al., forthcoming).

For the participants in the present study, overcoming legal obstacles was the point of departure in their narratives of how they were conceived. In contrast, when they talked about how they experienced growing up in an un-normative family, their narratives contained only few descriptions of legal obstacles. Social and legal parenthood were not congruent during their childhood, as the participants had at least one social parent who was excluded from legal parenthood during their childhood. However, the participants generally emphasized that the absence of legal ties had not affected their everyday lives. Elsewhere there have been descriptions of how parents in similar situations have drawn up wills and powers of attorney in order to secure their child’s right to inherit them, or to be able to make decisions about their child (Zetterqvist Nelson, 2007; Park et al., 2016; Malmquist and Spånberg Ekholm, 2020). One participant in the present study had been second-parent adopted, and additionally one mentioned his social parent’s wish to adopt him, but the remaining participants in the present study did not describe any such strategies. However, it is possible that they were not entirely aware of efforts potentially made by their parents to exert their parenthood. Indeed a few participants did mention that some situations might have been hard for their non-legal parent, but claimed that this had not affected them as children. In contrast, studies that focus on parental perspectives, and particularly those that focus on non-legally recognized gay or lesbian parents, generally depict non-legal parents’ huge frustration over not being able to achieve legitimate status as parent (Zetterqvist Nelson, 2007; Malmquist, 2015; Malmquist and Spånberg Ekholm, 2020). For the parents, being legally unrecognized may also lead to a feeling of being less of a parent (Bjärenstam and Dahlstedt, 2014; Henrikson and Sarelid, 2014).

It is interesting that the participants did not report inequalities between their parents as a result of differences in legal status. Rather, they emphasized the similarities in their emotional and social relationships with each of their parents. Further, they depicted parental authority as being unaffected by a lack of legal parenthood and guardianship. In contrast, previous studies of lesbian parents show that non-biological/non-legal parents often experience difficulties when negotiating their role both within the close family and in relation to society (Zetterqvist Nelson, 2007; Malmquist, 2015). That participants in the present study seldom reported such experiences could indicate that parents have been able to circumvent or compensate for potential deficiencies associated with the lack of legal parenthood to such an extent that these limitations were not noticeable to their children. Another potential interpretation is that the young adults were loyal toward their parents and, therefore, diminished any experiences of differences, as they did not want to hurt their non-biological parent(s). In fact several of the participants were uncertain which of their parents was a legal guardian, which further emphasized the limited role legal parenthood played in their experiences of growing up with same-sex parents.

An additional reason why legal parenthood had not been experienced as important is the common experience that people outside the family often did recognize social parenthood, e.g., all social parents had been invited to parent-teacher meetings. This highlights how schools can operate to legitimize social parenthood and normalize a variation of families, which in turn helps to make everyday life easier in these families. The finding contradicts Nordén’s (2018) interview study with young adults who grew up with LGBT parents during the same period as the current study. In Nordén’s study, problematic school interactions were prominent. These different findings may be a result of different samples or methodology. Nordén conducted in-depth interviews with a major focus on school experience and may therefore have been able to explore deficiencies in school to a larger degree. In contrast, our study had a primary focus on legislation, and contained no specific questions about school situations during childhood.

Despite the general experience that legal parenthood had had little impact, some participants recounted events where absence of legal parenthood had become strikingly apparent, such as in parental separations or deaths. These specific events pinpoint the cruciality of legal parenthood. Without the security of a legal tie, children may lose contact with their social parents, in the case of a conflictual divorce (e.g., Aylward and Alvelin, 2013; Gahan, 2019). One study of planned lesbian families in the United States showed how the likelihood of maintaining a relationship with the non-biological mother increased if the relationship had been legally secured through a second-parent adoption (Gartrell et al., 2011). None of the participants in the present study had lost contact with a parent, but one participant described how her mothers’ separation had led to her growing up far away from her non-legal mother and her sibling. This experience echoed the findings of Goldberg and Allen (2013), who found that several young adults whose lesbian mothers had separated had subsequently had only infrequent contact with their non-legal parent.

The death of a non-legal parent can present huge difficulties for offspring who have no right to inherit from that parent, or when siblings realize their different legal positions. Another type of event where legal parenthood had mattered was described by one participant who recounted an experience when his parents were in a conflict situation and their legal parent had been able to limit their non-legal parent’s position.

### Limitations and Implications

The present article reports results from a small-scale interview study. It is possible that a larger number of participants would have enriched the data and provided a deepened understanding of the topic. Another limitation of the study is that the adult participants’ reflections are most likely influenced by their own experiences in adulthood, thus, we do not know how they actually experienced legal parenthood while growing up.

While Swedish law today recognizes female same-sex parenting couples, this is still not the case in many other countries (ILGA, 2019). The present study shields light on the social consequences of legally unrecognized parent-child relations, which are important to acknowledge when legal reforms are called for elsewhere. The study also shows how previous lack of legal recognition continues to influence a cohort of people who were born prior to legislative changes, not the least by the time when life ends for their non-legal parents and inheritance is considered.

## Conclusion

The participants in the present study were born prior to the legal recognition of same-sex parenthood in Sweden. They depicted their own conception as the result of their parents’ navigating legal obstacles to find a pathway to parenthood, and indicated that “life had found its way” despite the limiting legislation. Although they had grown up with one or more non-legal parents, the lack of legal ties had, for most, not been experienced as problematic from their point of view as children. Only when the parents had divorced, or when one parent had passed away, had legal parenthood become crucial. Thus, most obstacles that legal limitations may have led to seem to have been handled by the adults without affecting the children. Lack of legal ties can be manageable, as long as good relationships are maintained and parents are still alive, but in the event of these difficult life events, (absence of) legal parenthood becomes a critical complicating factor.

## Data Availability Statement

The datasets generated for this study will not be made publicly available because of confidentiality.

## Ethics Statement

The studies involving human participants were reviewed and approved by Regionala etikprövningsnämnden, Linköping University. Written informed consent to participate in this study was provided by the participants’ legal guardian/next of kin. Requests to access the datasets should be directed to the corresponding author.

## Author Contributions

SA and JS have jointly constructed the interview guide, collected the data, transcribed the interviews, coded the data, made the initial analysis, and contributed to the writing of the final manuscript. AM planned the study, supervised SA and JS in their work, developed the analysis for the manuscript, and led the writing of the manuscript.

## Conflict of Interest

The authors declare that the research was conducted in the absence of any commercial or financial relationships that could be construed as a potential conflict of interest.

## References

[B1] Andersson K. Högstedt S. (2002). *Familj i Barnaögon, Sjätteklassares åsikter om Familj och Familjebildning.* Candidate thesis, Lunds universitet, Socialhögskolan vid Lunds universitet, Lund.

[B2] Andersson S. Salomonsson J. (2018). *Ungas Perspektiv på Föräldraskap och Ursprung: Reflektioner från Unga Vuxna Tillkomna Genom Könscellsdonation och/eller uppvuxna i regnbågsfamiljer.* Masters’ thesis, Linköping University, Linköping.

[B3] Anyan S. E. Pryor J. (2002). What is in a family? Adolescent perceptions. *Child. Soc.* 16 306–317. 10.1002/chi.716

[B4] Aylward A. Alvelin C. (2013). *Förlorande Barn.* Gothenburg: Copula Förlag.

[B5] Bäck-Wiklund M. Bergsten B. (1997). *Det Moderna Föräldraskapet: En Studie av Familj och kön i Förändring.* Stockholm: Natur & kultur.

[B6] Bergcrona L. Krantz M. (2014). *En Familj är två eller en vuxna. Och sen barn: En Tematisk Analys av hur barn till Frivilligt Ensamstående Mammor och Barn till Olikkönade Sammanboende Föräldrapar Pratar om Familj.* Masters’ thesis, Linköpings universitet, Institutionen för beteendevetenskap och lärande, Linköping.

[B7] Biblarz T. Stacey J. (2010). How does the gender of parents matter? *J. Marriage Fam.* 72 3–22. 10.1111/j.1741-3737.2009.00678.x

[B8] Bjärenstam N. Dahlstedt E. (2014). *”.att Ansöka om en Adoption till Sitt Eget Barn.” - En Kvalitativ Studie om Samkönat Föräldraskap och Närståendeadoption.* Candidates’ thesis, Lund university, Lund.

[B9] Bos H. M. Van Balen F. (2008). Children in planned lesbian families: Stigmatisation, psychological adjustment and protective factors. *Cult. Health Sex.* 10 221–236. 10.1080/13691050701601702 18432422

[B10] Braun V. Clarke V. (2006). Using thematic analysis in psychology. *Qual. Res. Psychol.* 3 77–101. 10.1191/1478088706qp063oa 32100154

[B11] Burr V. (2003). *Social Constructionism.* London: Routledge.

[B12] Cocker C. Hafford-Letchfield T. Ryan P. Barran C. (2019). Positioning discourse on homophobia in schools: what have lesbian and gay families got to say? *Qual. Soc. Work* 18 800–817. 10.1177/1473325018767720

[B13] Côté I. Lavoie K. (2019). A child wanted by two, conceived by several: lesbian-parent families negotiating procreation with a known donor. *J. GLBT Fam. Stud.* 15 165–185. 10.1080/1550428x.2018.1459216

[B14] Crichton M. Koepp D. Kennedy K. Molen G. R. Spielberg S. (1993). *Jurassic Park [Motion Picture].* Amblin Entertainment: United States.

[B15] Dannesboe K. I. (2016). “Ambiguous involvement: children’s construction of good parenthood,” in *Doing Good Parenthood Ideals and Practices of Parental Involvement*, eds Sparrman A. Westerling A. Lind J. Dannesboe K. I. (Basingstoke: Palgrave macmillian), 65–75. 10.1007/978-3-319-46774-0_6

[B16] Epstein R. Idems B. Schwartz A. (2013). Queer spawn on school. *Confero* 1 173–209.

[B17] Evertsson M. Jaspers E. Moberg Y. (forthcoming). “Parentalization of same-sex couples: family formation and leave rights in five northern European countries,” in *The Palgrave Handbook of Family Policy*, eds Nieuwenhuis R. Van Lancker W. (Basingstoke: Palgrave Macmillan).

[B18] Frisk Kockum K. Grönbäck M. (2018). *Kärlek är tjockare än blod” –Regnbågs- och donatorbarn reflekterar kring föräldraskap, biologiskt ursprung. (och)familjejuridik.* Masters’ thesis, Gothenburg University, Gothenburg.

[B19] Gahan L. (2019). Separation and post-separation parenting within lesbian and gay co-parenting (guild parented) families. *Aust. New Zealand J. Fam. Ther.* 40 98–113. 10.1002/anzf.1343

[B20] Gartrell N. Bos H. Koh A. (2019). Sexual attraction, sexual identity, and same-sex sexual experiences of adult offspring in the U.S. National Longitudinal Lesbian Family Stud. *Arch. Sex. Behav.* 48 1495–1503. 10.1007/s10508-019-1434-5 30888553

[B21] Gartrell N. Bos H. Peyser H. Deck A. Rodas C. (2011). Family characteristics, custody arrangements, and adolescent psychological well- being after lesbian mothers break up. *Fam. Relat.* 60 572–585. 10.1111/j.1741-3729.2011.00667.x

[B22] Goldberg A. E. Allen K. R. (2013). Donor, dad, or…? Young adults with lesbian parents’ experiences with known donors. *Fam. Process* 52 338–349. 23763691 10.1111/famp.12029

[B23] Golombok S. (2015). *Modern Families: Parents and Children in New Family Forms.* Cambridge: Cambridge University Press.

[B24] Golombok S. Spencer A. Rutter M. (1983). Children in lesbian and single-parent households: Psychosexual and psychiatric appraisal. *J. Child Psychol. Psychiatry* 24 551–572. 10.1111/j.1469-7610.1983.tb00132.x 6630329

[B25] Golombok S. Tasker F. Murray C. (1997). Children raised in fatherless families from infancy: Family relationships and the socioemotional development of children of lesbian and single heterosexual mothers. *J. Child Psychol. Psychiatry Allied Discipl.* 38 783–791. 10.1111/j.1469-7610.1997.tb01596.x 9363577

[B26] Henrikson M. Sarelid C. (2014). *Kanske, eventuellt, får jag vara förälder till mitt eget barn.* Cadidates’ thesis, Malmö university, Malmö.

[B27] ILGA (2019). *Map: Sexual Orientation Laws in the World.* Geneva: ILGA.

[B28] James A. (1999). “Parents: A children’s perspective,” in *What is a Parent: A Socio-Legal Analysis*, eds Bainham A. Sclater S. D. Richards M. (Oxford: Hart), 181–196.

[B29] Kuvalanka K. Leslie L. Radina R. (2014). Coping with sexual stigma: emerging adults with lesbian parents reflect on the impact of heterosexism and homophobia during their adolescence. *J. Adolesc. Res.* 29 241–270. 10.1177/0743558413484354

[B30] Lick D. Patterson C. Schmidt K. (2013). Recalled social experiences and current psychological adjustment among adults reared by gay and lesbian parents. *J. GLBT Fam. Stud.* 9 230–253. 10.1080/1550428x.2013.781907

[B31] Mägi E. Zimmerman L. (2015). *Stjärnfamiljejuridik: Svensk Familjelagstiftning ur ett Normkritiskt Perspektiv.* Malmö: Gleerups Utbildning.

[B32] Malmquist A. (2015). *Pride and Prejudice: Lesbian Families in Contemporary Sweden.* Doctral thesis, Linköping university, Linköping.

[B33] Malmquist A. (2016). *Lesbiska småbarnsföräldrar: Utmaningar i en tid av möjligheter.* Gothenburg: Makadam.

[B34] Malmquist A. (2018). “Bilaga 2. Barn och ungas perspektiv på föräldraskap och ursprung: Underlag för utredningen om faderskap och föräldraskap,” in *SOU 2018:68 Nya regler om faderskap och föräldraskap*, ed. Jungstedt S. (Stockholm: Nordstedts Juridik), 367–390.

[B35] Malmquist A. Spånberg Ekholm A. (2020). Swedish gay men’s pursuit of fatherhood: legal obstacles and strategies for coping with them. *Lambda Nordica* 24 53–80. 10.34041/ln.v24.580

[B36] Malmquist A. Zetterqvist Nelson K. (2008). Diskursiv diskriminering av regnbågsfamiljer: en analys av argument mot likställande av homo- och heterosexuellas föräldraskap. *Socialvetenskaplig Tidskrift* 15 315–331.

[B37] Malmquist A. Möllerstrand A. Wikström M. Zetterqvist Nelson K. (2014). ‘A daddy is the same as a mummy’: Swedish children in lesbian households talk about fathers and donors. *Childhood* 21 119–133. 10.1177/0907568213484342

[B38] Morgan D. (2011). *Rethinking Family Practices.* Basingstoke: Palgrave Macmillan.

[B39] Nordén P. (2018). *Regnbågsungar: Familj, Utbildning, Fritid.* Doctoral thesis, Gothenburg University, Gothenburg.

[B40] Park N. K. Kazyak E. Slauson-Blevins K. (2016). How law shapes experiences of parenthood for same-sex couples. *J. GLBT Fam. Stud.* 12 115–137. 10.1080/1550428x.2015.1011818

[B41] Pascal C. Bertram T. (2009). Listening to young citizens: the struggle to make real a participatory paradigm in research with young children. *Eur. Early Childh. Educ. Res. J.* 17 249–262. 10.1080/13502930902951486

[B42] Reczek C. (2020). Sexual- and gender-minority families: a 2010 to 2020 decade in review. *J. Marriage Fam.* 82, 300–325. 10.1111/jomf.12607 PMC7710266 33273747

[B43] Rigg A. Pryor J. (2007). Children’s perceptions of families: what do they really think? *Child. Soc.* 1 17–30.

[B44] Ryan-Flood R. (2009). *Lesbian Motherhood: Gender, Families and Sexual Citizenship.* Basingstoke: Palgrave Macmillan.

[B45] Skoog Svanberg A. Sydsjö G. Lampic C. (2019). Psychosocial aspects of identity-release gamete donation: Perspectives of donors, recipients, and offspring. *Upsala J. Med. Sci.* (in press). 10.1080/03009734.2019.1696431 PMC7720987 31802698

[B46] Slutsky J. Jadva V. Freeman T. Persaud S. Steele M. Steele H. (2016). Integrating donor conception into identity development: adolescents in fatherless families. *Fertil. Steril.* 106, 202–208. 10.1016/j.fertnstert.2016.02.033 27012652 PMC4922596

[B47] SOU (2001). *2001:10. Barn i homosexuella familjer. Public report from Swedish ministry of Justice.* Gujarat: SOU.

[B48] Štambuk M. Tadić Vujčić M. Maričić M. (2019). Pathways to parenthood among LGBTIQ people in Croatia: who wants to become a parent and how? *Revija za sociologiju.* 49 175–203. 10.5613/rzs.49.2.3

[B49] Tasker F. Granville J. (2011). Children’s views of family relationships in lesbian-led families. *J. GLBT Fam. Stud.* 7 182–199. 10.1080/1550428x.2011.540201

[B50] Tinnfält A. Jensen J. Eriksson C. (2015). What characterises a good family? Giving voice to adolescents. *Int. J. Adolesc. Youth* 20 429–441. 10.1080/02673843.2015.1018283

[B51] Tjora A. (2012). *Från Nyfikenhet till Systematisk Kunskap: Kvalitativ Forskning i Praktiken.* Lund: Studentlitteratur.

[B52] van Rijn-van Gelderen L. Bos H. M. W. Gartrell N. K. (2015). Dutch adolescents from lesbian-parent families: how do they compare to peers with heterosexual parents and what is the impact of homophobic stigmatization? *J. Adolesc.* 40 65–73. 10.1016/j.adolescence.2015.01.005 25658718

[B53] Vanfraussen K. Ponjaert-Kristoffersen I. Brewaeys A. (2002). What does it mean for youngsters to grow up in a lesbian family created by means of donor insemination? *J. Reproduct. Infant Psychol.* 20 237–252. 10.1080/0264683021000033165 16180285

[B54] Zadeh S. Ilioi E. C. Jadva V. Golombok S. (2018). The perspectives of adolescents conceived using surrogacy, egg or sperm donation. *Hum. Reprod.* 33 1099–1106. 10.1093/humrep/dey088 29701833 PMC5972639

[B55] Zetterqvist Nelson K. (2001). *Döttrar, söner och Homosexuella Föräldrar: En Studie av Homosexualitet sett ur barns och Föräldrars Perspektiv. Appendix 4, in SOU 2001:10. Barn i homosexuella familjer. Public report from Swedish ministry of Justice.* Gujarat: SOU.

[B56] Zetterqvist Nelson K. (2007). *Mot Alla Odds: Regnbågsföräldrars Berättelser om att Bilda Familj och få barn.* Malmö: Liber.

